# Supplementary material for: Colorimetric Test for Fast Detection of SARS-CoV-2 in Nasal and Throat Swabs
Source: ACS Sens. 2020 Sep 29;5(10):3043–8. doi: 10.1021/acssensors.0c01742 (PMC7534800; doi:10.1021/acssensors.0c01742)
Supplement: Supplementary file 1 — se0c01742_si_001.pdf [file se0c01742_si_001.pdf]

Colorimetric Test for Fast Detection of SARS-CoV-2 in Nasal and Throat Swabs

Bartolomeo Della Ventura,[1] Michele Cennamo,[2] Antonio Minopoli,[1] Raffaele Campanile,[1] Sergio Bolletti Censi,[3] Daniela Terracciano,[2] Giuseppe Portella,[2]* Raffaele Velotta[1]*

[1]Dipartimento di Fisica *Ettore Pancini* - Università di Napoli *Federico II*, Via Cintia, 26 Ed. 6 – 80126 Napoli (Italy)

[2]Dipartimento di Medicina Traslazionale - Università di Napoli *Federico II* - Via Pansini, 5 - 80131 Napoli (Italy)

[3]Cosvitec scarl - Via Galileo Ferraris, 171 - 80142 Napoli (Italy)

*Corresponding authors:

rvelotta@unina.it

portella@unina.it

S1. Materials

The tetrachloroauric acid trihydrate (code 520918) and sodium citrate (code W302600) were purchased by Sigma Aldrich. Antibodies used throughout the experiments were purchased by antibodiesonline.com company and were directed against spike proteins (anti-SARS-CoV-2 Spike S2 C-Term antibody, code ABIN1030641), membrane proteins (anti-SARS-CoV-2 Membrane Protein, SARS-CoV-2 M, code ABIN6952906) and envelope proteins (anti-SARS-CoV-2 Envelope, SARS-CoV-2 E N-Term antibody, code ABIN1031551) of the SARS-CoV-2 virus. MilliQ pure water was drawn from the Millipore water generator.

S2. Instruments

The centrifuge was from Thermo Scientific model Pico17.

UV−Vis spectroscopy and dynamic light scattering (DLS) were used to characterize both bare and anti-SARS-CoV-2 functionalized AuNPs. The UV−Vis extinction spectra were recorded by a Jenway 6715 UV/Vis spectrophotometer with 0.1 nm resolution and 0.2 nm spectral bandwidth. DLS measurements were carried out by using a Zetasizer Nano ZS (Malvern Instruments) equipped with a 633 nm He−Ne laser and an avalanche photodiode detector placed at the detection angle of 173°. The device was also able to perform ζ-potential measurements based on laser Doppler microelectrophoresis.

Transmission electron microscopy (TEM) micrographs were collected by a FEI Tecnai G2 S-twin apparatus operating at 200 kV (LaB6 source). The particle powder samples were transferred on carbon-coated copper grids (200 mesh) by dispersing them in ethanol and, then, by adding a drop on the copper grid and evaporating the solvent.

GloMax-Multi Microplate Reader from Promegawas used to measure the 96 multi-well plate at 560 nm in less than 1 minute.

S3. Gold nanoparticle synthesis

The gold nanoparticles (AuNP)s were synthesized by modifying an existing protocol.[1] We used $HAuCl_4$ (tetrachloroauric acid trihydrate) with a concentration of 10 mg / mL and concentrated sodium citrate (80 mM). 1 mL of $HAuCl_4$ was spiked in 100 mL of MilliQ under vigorous stirring until boiling. The beaker containing the solution was cleaned with *aqua regia* to avoid salt contamination. After boiling, 2 mL of sodium citrate was added into the solution keeping the same temperature and the vigorous stirring for 20 minutes. Then, when the colour of the solution changed becoming bright red, the beaker was moved on a cold plate, by keeping the stirring for 2 hours. This synthesis time was found to be indispensable in order to obtain a good monodisperse and homogeneous AuNP distribution. The optical density of approximately 0.6 at 450 nm allowed us to

estimate the AuNP concentration as 6×10^{10} AuNPs/mL.[2] Afterward, the AuNP colloidal solution was stored in dark conditions at 4°C.

S4. Optical and morphological AuNP characterization

Figure S1a shows the extinction spectrum of naked AuNPs, in which the LSPR peak at 523 nm is clearly visible as expected for 20 nm diameter spherical AuNPs. Size and shape of AuNPs were also characterized by TEM images (an example is reported in Figure S1b), which highlighted the presence of quite regular spherical monodisperse nanoparticles with a diameter of approximately 20 nm. The DLS measurements (Figure S1c) confirmed the mono-dispersion of the synthetized colloidal solution. ζ-potential measurements (Figure S1d) were performed to verify the surface charge of the nanoparticles that is approximately of (-30 ± 1) mV thereby confirming the existence of electrostatic repulsion forces among the particles that prevent their aggregation.

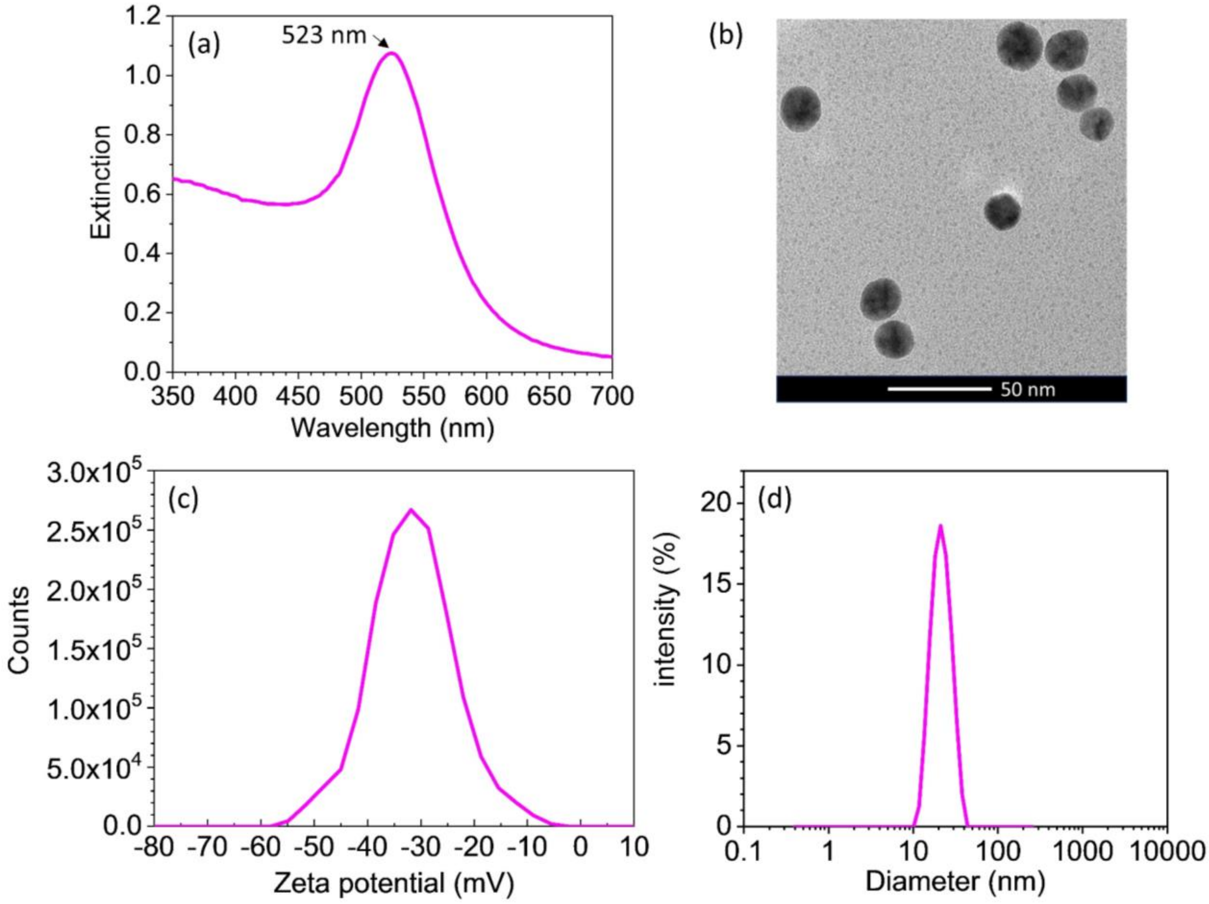

Figure S1. (a) Extinction spectrum of naked AuNP colloidal solution (b) TEM image of naked AuNPs. (c) Zeta potential distribution of naked AuNPs. (d) Intensity-size distribution of naked AuNPs.

S5. Functionalization

The functionalization of the AuNPs was achieved by Photochemical Immobilization Technique (PIT). The UV source (Trylight lamp, Promete S.r.l., Figure S2a) consisted of two U-shaped low-pressure mercury lamps (6 W at 254 nm), in which a standard quartz cuvette could be easily housed (Figure S2b). By considering the wrapping geometry of the lamps and the proximity of the cuvette, the irradiation intensity used for the thiol group production was approximately 0.3 W/cm^2. Such an intensity is high enough to warrant an effective production of specific thiol groups, while avoiding any significant photolysis of the disulphide bridge that poorly absorbs at 254 nm.[3]

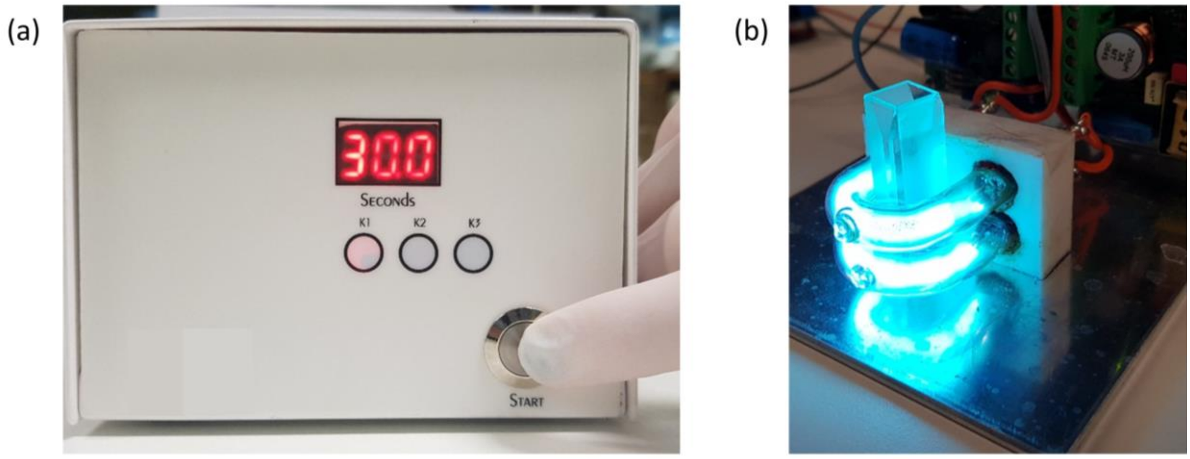

Figure S2. (a) Trylight lamp used to irradiate the antibodies. (b) Inside of the Trylight lamp where the UV source is visible.

The activation of a single polyclonal antibody (pAb) was achieved by irradiating a solution of 100 µL [10 µg pAb/mL] for 30 s. The activated solution of pAb was spiked into a colloidal of AuNPs so to achieve their functionalization against each of three surface proteins (spike, membrane, and envelope). Despite from preliminary measurements we noticed that combinations of antibodies seemed to suggest that anti-M plays a quite minor role, whereas anti-S enhanced the fundamental contribution from anti-E, the difficulty to deal with infected samples prevented us from optimizing the ratio among them. Thus, the three solutions of functionalized AuNPs were subsequently mixed with 1:1:1 ratio so to maximize the chance to cover the virion surface. Such a mix of functionalized AuNPs (f-AuNPs) was used throughout this study.

As shown in Figure S3a, the AuNP functionalization involved a red shift of the LSPR peak of 3.8 nm. Such a behaviour is due to the change of the refractive index surrounding the nanoparticle. It is worth noticing that PIT ensured a robust close-packing arrangement of pAb onto the AuNP surface as demonstrated by the lack of any change of the extinction spectrum after the centrifugation at 3.5 g for 30 min and the BSA (bovine serum albumin) [25 µg/mL] blocking.

Figure S3b shows the kinetic study aimed at finding the best concentration of the IgGs to functionalize the AuNPs. The functionalization was achieved by adding 100 µL of irradiated Ab solution at several

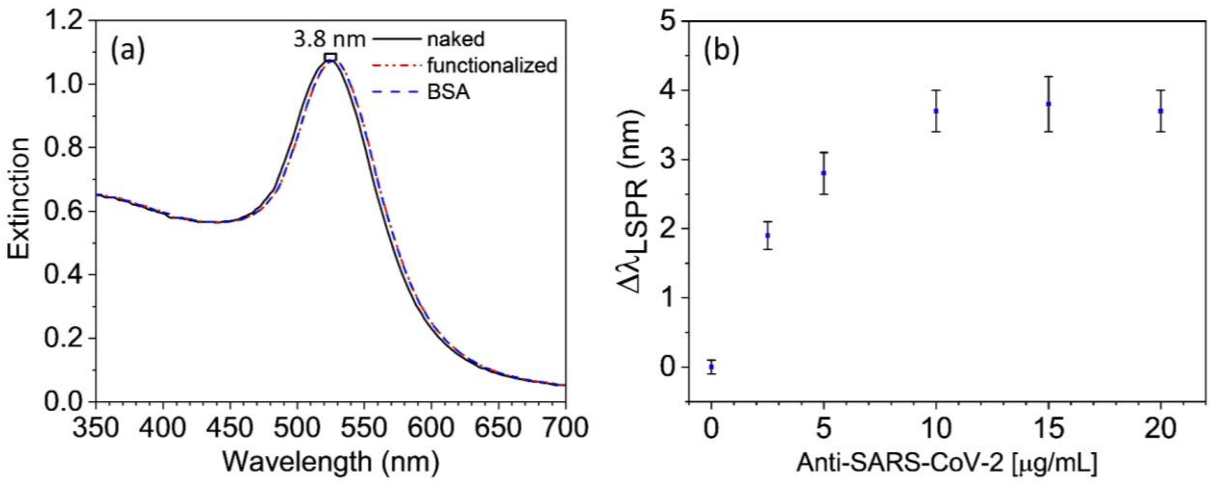

Figure S3. (a) Extinction spectra of naked (black continuous line), functionalized (dashed-dotted red line) and blocked (dashed blue line) AuNPs. The slight red shift of 3.8 nm is due to the presence of a thin dielectric layer surrounding AuNPs after functionalization by IgGs. No change after blocking step demonstrates the achievement of a close-packing arrangement of Abs onto gold surface. (b) Red shift of the LSPR peak as a function of anti-SARS-CoV-2 concentration. The saturation for 100 mL of AuNPs with OD ~ 1.0 was reached with a concentration of 10 µg/mL of Abs.

concentrations into 100 mL of AuNPs (OD ~ 1.0). Such a volume was added in 5 spikes (20 µL each one) followed by gentle stirring to avoid AuNPs aggregation. The LSPR peak red-shifted as the anti-SARS-CoV-2 increased up to 10 µg/mL that corresponded to a maximum red shift of 3.8 nm. For larger amounts of anti-SARS-CoV-2, AuNP surface was fully covered and no change in LSPR wavelength was detectable.

S6. Storage of the samples and validation of the measurements with PCR

Naso-oropharyngeal swab specimens in 3-ml Universal transport medium (UTM, Copan Brescia, Italia) were collected in the Infectious Diseases Unit of University Hospital Federico II (Naples, Italy) and tested for the presence of SARS-CoV-2 RNA within 24 h of collection. We used Abbott Real Time SARS-CoV-2 assay, a dual target assay for RdRp and N-genes. The two SARS-CoV-2-specific probes are labeled with the same fluorophore whereas the internal control (IC)-specific probe is labeled with a different fluorophore, allowing simultaneous detection of both SARS-CoV-2 and IC amplified products in the same reaction well. The Abbott RealTime SARS-CoV-2 assay were performed on the Abbott m2000 System. Total nucleic acids were extracted from UTM using an input sample volume of 600 µL. Amplification was performed following manufacturer's instructions. Swabs were stored at -80° before further assays.

S7. Threshold cycle in PCR measurement and viral load

A real-time PCR assays detects a positive sample by the accumulation of a fluorescent signal indicating the amplification of the target sequence. The cycle threshold is defined by the number of cycles required for the

fluorescent signal to cross the background level (threshold). C_t levels are inversely proportional to the amount of target nucleic acid in the sample: the lower C_t the larger the amount of target sequence).

In SARS-CoV-2 infection it is possible to distinguish two different phases: early in the course of infection (both symptomatic and asymptomatic) viral loads are high, ranging 10,000 to several million copies/mL and resulting in low C_t readouts by real-time PCR. The C_t number can vary according to the sensitivity of real-time PCR; for instance, in the early phases of the infection the Abbott SARS-CoV-2 device typically gives a $12<C_t<15$. Afterwards, the viral load decreases by several orders of magnitude resulting in a higher C_t (>30). It is important to point out that real-time PCR assay with low sensitivity might not detect these samples.

S8. FDTD optical simulations

The optical response of the proposed immunosensor was simulated by "FDTD solutions" tool implemented in Lumerical software that delivers numerical solutions of the Maxwell's equations by finite-difference time-domain (FDTD) method within a Mie problem-like workspace. The system was investigated by using a linearly polarized electromagnetic radiation and the photons transmitted through the plasmon object were collected by a photodetector positioned on the opposite side of the workspace to retrieve its extinction spectrum.

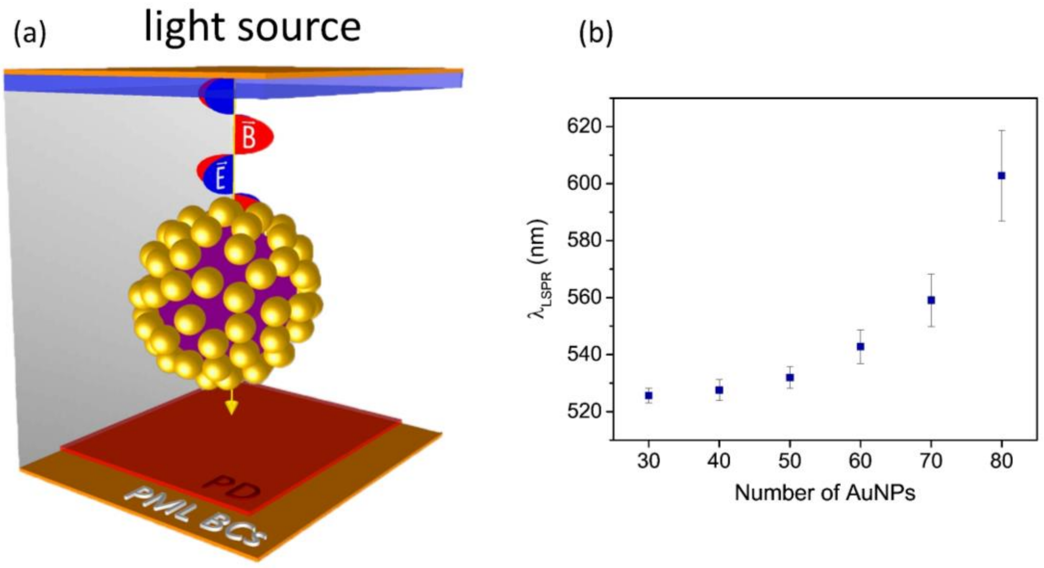

Figure S4. (a) Lumerical workspace used throughout the optical simulations. (b) LSPR wavelength as a function of the AuNP number surrounding the 100 nm diameter dielectric sphere. Due to the randomness of the AuNP positioning onto the virus surface, the simulations were conducted by running the code ten times and, hence, the data are shown as mean value ± SD.

Symmetric/anti-symmetric boundary conditions (BCs) were set along *x* and *y* direction to reduce the simulation time by a factor 8 without worsening the result accuracy, whereas perfect matched layer BCs were set in *z* direction to guarantee the total absorption of the electromagnetic radiation both backscattered through the light source plane and travelling behind the photodetector. The workspace was discretized over a mesh with 0.5 nm spatial resolution, a value small enough to warrant high accuracy while keeping the simulation time within few

hours. The AuNPs were modelled as 20 nm diameter gold spheres[4] while the virion was represented as a 100 nm diameter homogeneous dielectric sphere[5] of 1.45 refractive index[6]. AuNPs were randomly positioned on the virion surface. A schematic representation of the simulation workspace is depicted in Figure S4a, whereas Figure S4b shows the LSPR wavelength as a function of the number of AuNPs positioned onto the virion surface.

References

(1) Pollitt, M. J.; Buckton, G.; Piper, R.; Brocchini, S. Measuring Antibody Coatings on Gold Nanoparticles by Optical Spectroscopy. *RSC Adv.* **2015**, *5* (31), 24521–24527. https://doi.org/10.1039/C4RA15661G.

(2) Amendola, V.; Meneghetti, M. Size Evaluation of Gold Nanoparticles by UV-Vis Spectroscopy. *J. Phys. Chem. C* **2009**. https://doi.org/10.1021/jp8082425.

(3) Della Ventura, B.; Banchelli, M.; Funari, R.; Illiano, A.; De Angelis, M.; Taroni, P.; Amoresano, A.; Matteini, P.; Velotta, R. Biosensor Surface Functionalization by a Simple Photochemical Immobilization of Antibodies: Experimental Characterization by Mass Spectrometry and Surface Enhanced Raman Spectroscopy. *Analyst* **2019**, *144* (23), 6871–6880. https://doi.org/10.1039/C9AN00443B.

(4) Haynes, W. M. *CRC Handbook of Chemistry and Physics*; Haynes, W. M., Lide, D. R., Bruno, T. J., Eds.; CRC Press, 2016. https://doi.org/10.1201/9781315380476.

(5) Zhu, N.; Zhang, D.; Wang, W.; Li, X.; Yang, B.; Song, J.; Zhao, X.; Huang, B.; Shi, W.; Lu, R.; Niu, P.; Zhan, F.; Ma, X.; Wang, D.; Xu, W.; Wu, G.; Gao, G. F.; Tan, W. A Novel Coronavirus from Patients with Pneumonia in China, 2019. *N. Engl. J. Med.* **2020**, *382* (8), 727–733. https://doi.org/10.1056/NEJMoa2001017.

(6) Ma, L.; Zhu, S.; Tian, Y.; Zhang, W.; Wang, S.; Chen, C.; Wu, L.; Yan, X. Label-Free Analysis of Single Viruses with a Resolution Comparable to That of Electron Microscopy and the Throughput of Flow Cytometry. *Angew. Chemie Int. Ed.* **2016**, *55* (35), 10239–10243. https://doi.org/10.1002/anie.201603007.
